# Endoleak Detection after Endovascular Aortic Repair via Coded-Excitation Ultrasound—A Feasibility Study

**DOI:** 10.3390/jcm12113775

**Published:** 2023-05-31

**Authors:** Amun G. Hofmann, Fabian R. Klosz, Irene Mlekusch, Georg Wickenhauser, Corinna Walter, Afshin Assadian, Fadi Taher

**Affiliations:** Department of Vascular and Endovascular Surgery, Klinik Ottakring, 1160 Vienna, Austria

**Keywords:** B-Flow, ultrasound, imaging, EVAR, endovascular aortic repair, endoleak

## Abstract

Endoleaks are the most common complication after endovascular aortic repair (EVAR). Their correct identification is one of the main objectives of surveillance protocols after EVAR. So far, computed tomography angiography (CTA), contrast-enhanced (CEUS) and Duplex ultrasound (DUS), as well as magnetic resonance angiography, have been investigated for their ability to detect endoleaks. In general, all technologies have distinct benefits and disadvantages, with CTA and CEUS emerging as the reference standard for surveillance after EVAR. However, they are both contrast-enhancer-dependent, and CTA additionally exposes patients to ionizing radiation. In the present study, we investigated B-Flow, a type of coded-excitation ultrasound that was specifically designed to optimize the visualization of blood flow, for its ability to detect endoleaks, and compared its performance to CEUS, CTA, and DUS. In total, 34 patients were included in the analysis that accumulated in 43 distinct B-Flow investigations. They underwent a total of 132 imaging investigations. Agreement between B-Flow and other imaging modalities was high (>80.0%), while inter-method reliability can be interpreted as good. However, with B-Flow, six and one endoleaks would have been missed compared to CEUS and CTA, respectively. Regarding endoleak classification, all metrics were lower but retained an adequate level of comparison. In a subset of patients requiring intervention, B-Flow had 100% accuracy regarding both endoleak detection and classification. Ultrasonography enables endoleak detection and classification without the need for pharmaceutical contrast enhancement or radiation. Ultrasound coded-excitation imaging in the application of B-Flow could further simplify surveillance after EVAR by offering adequate accuracy without requiring intravenous contrast enhancement. Our findings may promote subsequent investigations of coded-excitation imaging for endoleak detection and classification in the surveillance after EVAR.

## 1. Introduction

Endovascular aneurysm repair (EVAR) as a treatment option for (infrarenal) abdominal aortic aneurysm (AAA) has established itself as a valuable therapeutic strategy since its introduction in the mid-1980s [1]. Compared to open surgical reconstruction (OSR), EVAR incorporates several advantages, including improved peri-operative survival [2] and higher cost effectiveness [3]. However, absent long-term survival benefits and higher re-intervention and rupture rates [4] counterbalance its advantages and require individualized decision making in the planning stages to develop a preferred treatment strategy for each case [5]. In the acute setting of (suspected) ruptured AAA, a partial inversion of advantages has been observed. Short-term survival is comparable between EVAR and OSR [6], whereas long-term mortality rates are lower after endovascular treatment [7]. Additionally, patients can be discharged earlier after EVAR [6], and have similar levels of re-interventions, while its impact on cost effectiveness is analogous to unruptured cases [7]. In summary, EVAR is often considered the preferred treatment modality for ruptured AAA under the limitation of permitting anatomy and center expertise [5].

EVAR requires subsequent surveillance at higher intervals compared to OSR due to a set of potential complications inherent to the procedure, including in-stent thrombosis and mechanical complications associated with the prosthesis, as well as leakage with persisting blood flow into the aneurysm sac [8]. Endoleaks are both the most common complication after EVAR and simultaneously the most common indication for secondary interventions [9]. Persisting feeding of the aneurysm sac effectively annuls the therapeutic success, and can lead to further AAA expansion and subsequent rupture [10]. Accordingly, endoleaks are therefore the most frequent cause of rupture following EVAR [11]. Endoleaks are commonly classified based on location and (dysfunctional) stent component, ranging from type I–IV endoleaks, with endotension occasionally being referred to as a type V endoleak [5,12].

Various imaging modalities have been investigated for surveillance in general, and endoleak detection specifically after EVAR with computed tomography angiography (CTA), repeatedly being discussed as the reference/gold standard [12,13]. This is based on its high resolution, image quality, and sensitivity to detect complications [12]. However, potential issues associated with pharmaceutical contrast enhancement [14] and cumulative exposure to ionizing radiation [15] are considerable limitations of CTA. Magnetic resonance imaging/angiography (MRA) eliminates concerns associated with radiation and iodinated contrast agents [16] and, additionally, it was reported to be more sensitive for endoleak detection (especially type II) in a meta-analysis [17]. Nevertheless, MRA is associated with its own limitations, including costs, availability, claustrophobic patients, or incompatible implants, such as pacemakers [13]. Duplex (DUS) and contrast-enhanced ultrasound (CEUS) have also been investigated for their ability to detect and classify endoleaks. While DUS has a high specificity, its sensitivity is low when compared to CTA [18]. CEUS has both high specificity and high sensitivity regarding endoleak detection [19] but is yet again contrast-agent-dependent. The matter is complex due to several factors that must be considered, and distinct benefits and limitations have to be weighed against each other, namely, radiation, contrast enhancement, accuracy, and cost effectiveness. This might be further complicated by the fact that different endoleak types are better displayed with certain imaging modalities. For example, type II endoleaks are well visualized with CEUS compared to CTA [20,21].

Recently, multiple efforts have been made to transition from contrast-medium-dependent imaging technologies for surveillance after EVAR. Most prominently, micro-vascular imaging using Doppler ultrasound technology developed by Toshiba has been shown to be effective, repeatable, and reliable when being compared to CEUS and CTA regarding endoleak detection [22,23,24].

Coded-excitation ultrasound is a technology that improves signal-to-noise ratio and therefore image quality [25]. B-Flow is a digitally encoded excitation ultrasound developed by GE Healthcare (Chicago, IL, USA) to optimize blood flow visualization [26]. It has been previously investigated for a plethora of different applications in clinical medicine [27]. In the present project, we investigated B-Flow’s efficacy in detecting and classifying endoleaks after EVAR compared to established imaging modalities as a drug- and radiation-independent alternative.

## 2. Materials and Methods

### 2.1. Patient Recruitment and Study Design

Following review board approval (ID: EK-20-007-VK, Ethics Committee of the City Government of Vienna), including a waiver of informed consent, a retrospective analysis of patients from a prospectively held database on endovascular aortic repair cases was performed. Only follow-up investigations including B-Flow ultrasound conducted at the department’s outpatient center were included in the study. Most patients in the present study had ultrasound investigations due to impaired kidney function or to further classify previously described endoleaks.

### 2.2. Computed Tomography

Computed tomography and CTA were performed by a radiologist either in-house or in external institutes. Computed tomography results without angiography were only used to assess and compare aneurysm diameters in the study. CTA was performed as a triple-phase study including unenhanced, arterial-contrast-enhanced, and delayed phases. CTA reviews for this study were routinely performed by radiologists and re-examined by a vascular surgeon in the outpatient clinic. Endoleaks were classified according to the current guidelines of the European Society of Vascular Surgery [5].

### 2.3. Ultrasound Examinations

Ultrasound examinations were performed using the Logiq S8 device (GE Healthcare, Chicago, IL, USA) by either a specialized vascular radio technician or a vascular diagnostic general practitioner. All imaging study results were then validated by a vascular surgeon. In a limited number of cases, the ultrasound studies were conducted by a radiologist. The routine workflow included subsequent ultrasound imaging studies starting with B-mode followed by DUS, B-Flow, and finally CEUS. Aneurysm diameter was exclusively measured in B-mode. CEUS was performed after intravenous Sonovue (Bracco, Milan, Italy) injection as a bolus followed by a flush of saline solution. Images and video clips of the examinations were stored locally on the ultrasound devices.

### 2.4. Statistical Analysis

Statistical analysis was performed with *R version 4.1.3 (One Push-Up)* (The R Foundation for Statistical Computing, Vienna, Austria) in *RStudio version 2022.02.2+485* (RStudio, PBC, Boston, MA, USA). Descriptive statistics were conducted in routine workflows. Averages are depicted as median (1st–3rd quartile) or mean (+/− standard deviation); proportions are given in percent. Comparisons regarding endoleak detection between imaging modalities and endoleaks were initially treated as a binary outcome (detected endoleak vs. no detected endoleak) and evaluated based on agreement and inter-method reliability. Agreement is expressed in percent, while inter-method reliability was assessed using Cohen’s kappa coefficient (kappa). Interpretation of kappa is based on Landis and Koch [28,29]. Subsequently, endoleak classification was evaluated analogously for detected type of endoleak (or combination of detected endoleaks in case of multiple findings).

## 3. Results

### 3.1. Participant Characteristics

In total, 34 patients were included. The sample consists of 31 men and 3 women. The mean age at surgery was 75.4 years. Mean body mass index at the time of surgery was 27.5 kg/m^2^. Primary procedures were conducted between 2009 and 2022. Patients underwent a total of 43 follow-up appointments at the out-patient clinic, resulting in 132 imaging studies. The follow-ups took place between January 2021 and December 2022. The majority of patients received EVAR (29), while two patients received branched EVAR, and three patients underwent fenestrated EVAR after standard EVAR (see Table 1). By default, since it was defined as an inclusion criterion, under the present study design, all 43 follow-ups included B-Flow. Additionally, the follow-ups comprised 18 CTA, 40 DUS, and 41 CEUS studies (see Table 2).

### 3.2. Aneurysm Diameter

**Figure 1:**
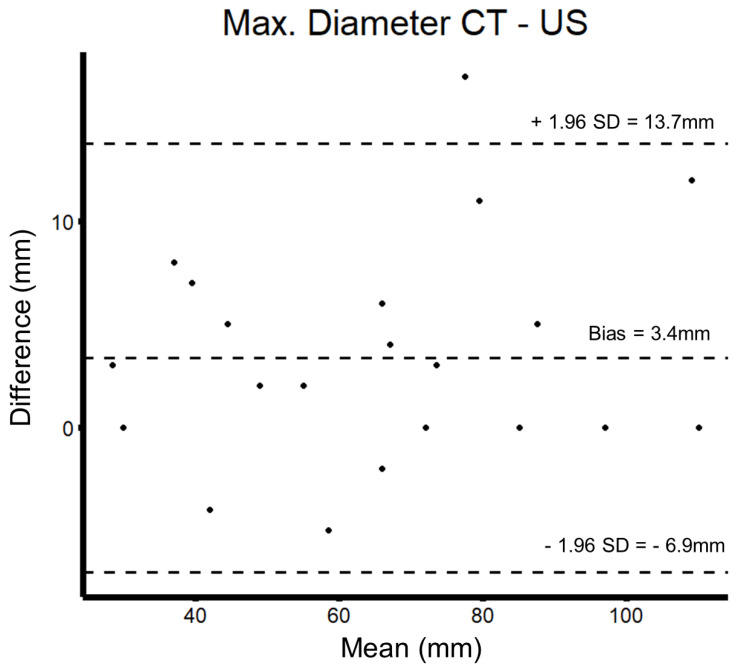
Bland–Altman plot of the maximal aneurysm diameter measured using computed tomography and ultrasound.

For matters of quality control, maximum aneurysm diameter, as measured using ultrasound and a CT scan, was compared to ensure the validity of the conducted ultrasound studies. In the present sample, 21 paired CT and ultrasound examinations were included (in 3 of these, patient CT without angiography was available). Mean maximum diameter was 69 mm in all CT studies and 63 mm in all ultrasound findings. The median delay between CT and ultrasound was 45 days (Q1–Q3: 11–84 days). Mean difference between imaging modalities was approximately 3 mm, and with the exception of one study, all fell within the confidence interval of the Bland–Altman plot (see Figure 1).

### 3.3. Endoleak Detection

**Figure 2:**
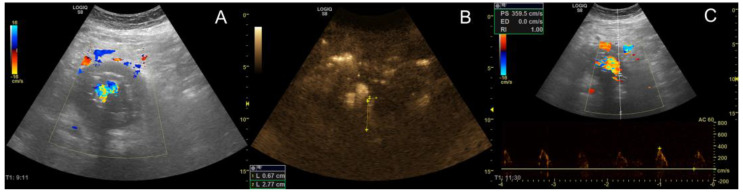
Ultrasound surveillance investigation after EVAR. While the lumbar type II endoleak could not be displayed via Duplex ultrasound (**A**), B-Flow allowed sufficient visualization (**B**) that could be validated with pulsed-wave Doppler (**C**).

**Figure 3:**
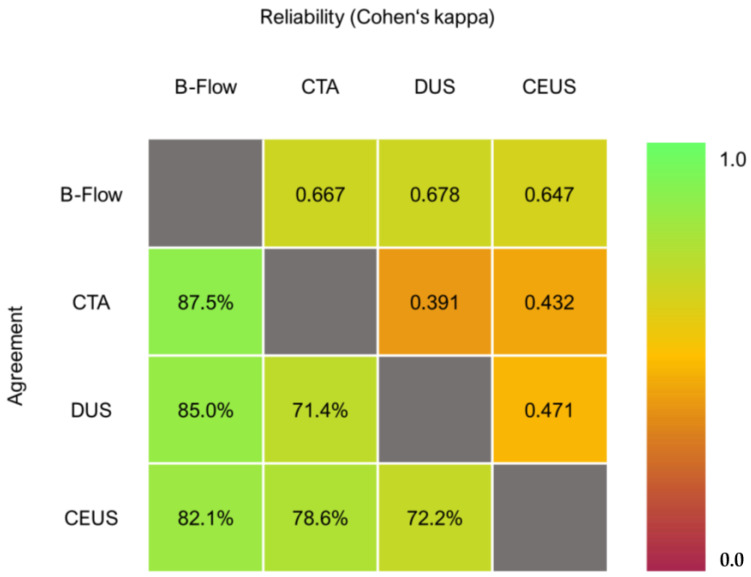
Agreement and reliability matrix regarding endoleak detection.

An example of an ultrasound surveillance investigation after EVAR is shown in Figure 2. B-Flow showed an agreement of 82.1%, 87.5%, and 85.0% with CEUS, CTA, and DUS, respectively. The interpretation of Cohen’s kappa is good when comparing B-Flow to all three imaging modalities. B-Flow had the highest agreement and reliability metrics in the sample, whereas DUS resulted in comparably low metrics in both regards (see Figure 3).

**Figure 4:**
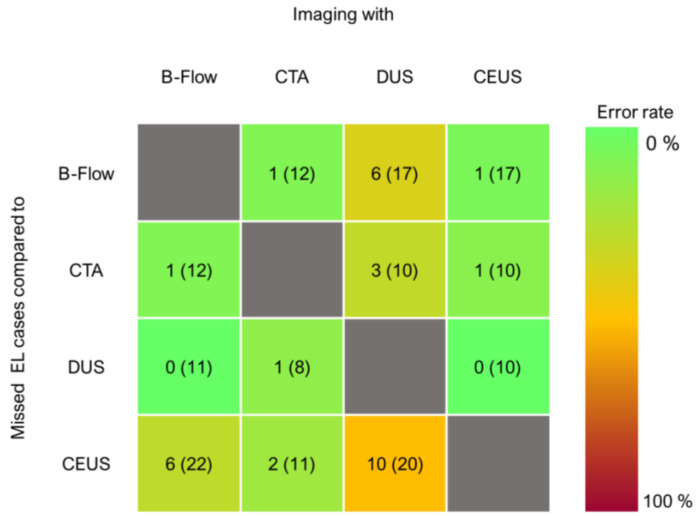
Figure 4 displays how many endoleaks would have been missed in a specific imaging modality compared to paired investigations with other technologies. Number in brackets is the sum of endoleaks found in the reference imaging modality in paired studies. The color gradient illustrates the relative error rate of the imaging technology of interest compared to the reference modality. (EL = endoleak).

Since agreement and reliability imply equal weights on diverging outcomes, i.e., missed endoleaks and additionally detected endoleaks affect the metrics in the same way, error rates regarding endoleak detection were further analyzed. B-Flow could not visualize 1 out of 12 endoleaks in paired CTAs and 6 out of 22 in paired CEUS investigations. In the present sample, no imaging modality had a 0% relative error rate. CEUS had the lowest relative error rates, while DUS had the highest (see Figure 4).

In total, B-Flow missed seven type II endoleaks compared to CEUS. However, in one case, a combined type II/type III was identified in CEUS, where the type III endoleak was visualized in B-Flow. Compared to CTA, B-Flow missed a type II endoleak and a type I endoleak in a combined type I/type II case, where B-Flow solely identified the type II endoleak.

### 3.4. Endoleak Classification

**Figure 5:**
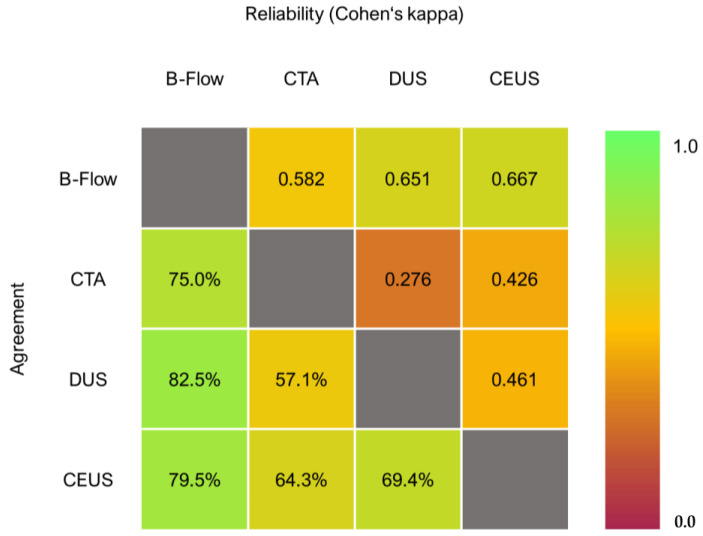
Agreement and reliability matrix regarding endoleak classification.

B-Flow showed an agreement of 79.5%, 75.0%, and 82.5% with CEUS, CTA, and DUS, respectively. The interpretation of Cohen’s kappa is good when comparing B-Flow to DUS and CEUS, and moderate when being evaluated against CTA. Yet again, B-Flow had the highest agreement and reliability metrics in the sample, whereas DUS resulted in comparably low metrics in both regards (see Figure 5).

Since missed endoleaks contribute to lowering agreement regarding endoleaks classification analogously to true differential classification, a subsequent comparative analysis was conducted filtering true differential classifications. In one case, after B-Flow investigation, we suspected a type IV endoleak that appeared as a type II endoleak in CTA. Both DUS and CEUS also displayed a type IV endoleak in the respective case. In all other investigations, B-Flow resulted in the same classification as the other imaging modalities.

### 3.5. Sensitivity Analysis

A sensitivity analysis was conducted by cross validating findings from imaging studies with endoleaks identified during subsequent interventional angiography. B-Flow correctly identified and classified all endoleaks that required treatment in the present sample (see Table 3).

## 4. Discussion

B-Flow showed strong agreement regarding endoleak detection compared to CTA, DUS, and CEUS (82.5–87.5%), as well as good inter-method reliability (following the interpretation proposed by Landis and Koch [28]). A more granular analysis of correct endoleak classification (including missed endoleaks) resulted in lower but still acceptable metrics. However, after removing endoleaks that could not be identified, B-Flow classifications showed 100% agreement with other types of ultrasound, and only one true discordant classification compared to CTA. We therefore conclude that B-Flow shows good agreement and reliability for both endoleak detection, as well as classification, compared to established imaging modalities after the endovascular treatment of aortic aneurysms.

However, the present study is an observational feasibility trial. Conclusions drawn from the conducted analyses should therefore be regarded as hypothesis-generating and require validation in subsequent trials. Apart from limitations regarding power based on the sample size and those inherent to the study design, generalizability needs to be investigated. Potential biases could arise from the single-center setting and absent proof-investigator independence. Even though it seems reasonable to assume that inter-rater differences in B-Flow are comparable to DUS [30], CEUS [31], or B-Flow itself in other clinical applications [32] and therefore acceptable, it also remains to be seen whether B-Flow shows similar performance regarding endoleak detection after more complex endovascular grafts. While the study included several cases of branched and fenestrated grafts, the sample size is too small to infer conclusions.

Previous studies often used CTA as a reference standard to evaluate CEUS and DUS. In a meta-analysis, the pooled sensitivity regarding endoleak detection was 0.74 for DUS and 0.96 for CEUS, while pooled specificity was 0.94 and 0.85, respectively [33]. However, in the present sample, every imaging modality would have missed endoleaks that were identified in other imaging studies, prompting the question of feasibility for sensitivity (true positive rate) and specificity (true negative rate) analyses, since they require knowledge of the true prevalence of a disease/outcome in the study population, which effectively might be unknown in the present case. Therefore, we also actively refrained from such analyses. Additionally, the necessity of CTA for surveillance after EVAR has been previously questioned in other works, with the consequential implications that CEUS might be enough to replace CTA [34,35]. Similarly, it was previously hypothesized that DUS might be sufficient for surveillance, since a lower sensitivity regarding endoleak detection compared to CTA is not necessarily clinically significant considering that DUS is able to reliably identify cases requiring secondary intervention [36]. The specific combination of CTA and CEUS for endoleak detection has also been proposed due to improved endoleak visualization (especially small ones) [37].

B-Flow inherently shares certain characteristics with other ultrasound technologies, most importantly its ability to provide angiodynamic imaging while providing adequate endoleak detection rates compared to DUS and CEUS. Whether it can replace radiation- and contrast-enhancer-dependent imaging, however, remains to be seen. In the present sample it proved to be more reliable than DUS. While it missed several endoleaks compared to CEUS and CTA, the clinical significance of it is questionable, since they were mostly type II endoleaks that do not necessarily require secondary intervention. All treated type II endoleaks were also correctly detected as well as classified by B-Flow. Furthermore, we hypothesize that in a combined imaging algorithm with CTA the technology might under certain circumstances be able to replace CEUS, eliminating the need for a dual contrast agent application.

In a clinical setting, future surveillance investigations could also be conducted in a modular workflow setting, starting with the least invasive imaging modality and prompting transition to more invasive methods in cases where the primary technology does not result in a sufficient study. For example, surveillance imaging could in the future be conducted primarily with B-Flow or also micro-vascular imaging, depending on the available device. In selected cases where these imaging technologies do not establish definite findings, contrast-enhancer-dependent examinations such as CEUS or CTA could be conducted. A modular workflow would also promote a transition from one-size-fits-all paradigms and introduce principles of precision medicine for the surveillance after endovascular interventions. Nevertheless, this requires the establishment of solid bodies of evidence for these investigational ultrasound technologies.

Coded excitation ultrasound implemented as B-Flow is a promising candidate that deserves to be further investigated for its use in surveillance after EVAR in general and for endoleak detection in specific.

## Figures and Tables

**Table 1:** Demographic and clinical characteristics of the study population. Age and BMI at surgery are given as median (Q1–Q3).

Baseline Characteristics	
N	34
Age at surgery (in years)	78.1 (73.8–78.3)
Sex (female:male)	3 (8.8%):31 (91.2%)
BMI (at surgery)	27.7 (24.2–31.4)
Follow-up appointments	43
Imaging studies	132
Graft type	
EVAR	29
BEVAR	2
FEVAR after failed EVAR	3

**Table 2:** Summary of conducted imaging studies and described endoleaks.

Follow-Ups	
**B-Flow**	
Examinations	43
With Endoleaks	19
**CTA**	
Examinations	18
With Endoleaks	12
**DUS**	
Examinations	40
With Endoleaks	11
**CEUS**	
Examinations	41
With Endoleaks	22

**Table 3:** Type of endoleak found in imaging, and subsequently identified (and treated) endoleak during interventional angiography.

	B-Flow	CEUS	CTA	Angiography
Case 1	Type II	Type II	Type II	Type II
Case 2	Type II	Type II	-	Type II
Case 3	Type II	-	-	Type II
Case 4	Type II	Type II	-	Type II

## Data Availability

Data can be made available upon reasonable request to the corresponding author.
